# Selective Phenotyping, Entropy Reduction, and the Mastermind game

**DOI:** 10.1186/1471-2105-12-406

**Published:** 2011-10-20

**Authors:** Julien Gagneur, Markus C Elze, Achim Tresch

**Affiliations:** 1 European Molecular Biology Lab, 69122 Heidelberg, Germany; 2 Johannes Gutenberg-University, 55099 Mainz, Germany; 3 Gene Center, Ludwig-Maximilians-University, 81377 Munich, Germany

## Abstract

**Background:**

With the advance of genome sequencing technologies, phenotyping, rather than genotyping, is becoming the most expensive task when mapping genetic traits. The need for efficient selective phenotyping strategies, *i*.*e*. methods to select a subset of genotyped individuals for phenotyping, therefore increases. Current methods have focused either on improving the detection of causative genetic variants or their precise genomic location separately.

**Results:**

Here we recognize selective phenotyping as a Bayesian model discrimination problem and introduce SPARE (Selective Phenotyping Approach by Reduction of Entropy). Unlike previous methods, SPARE can integrate the information of previously phenotyped individuals, thereby enabling an efficient incremental strategy. The effective performance of SPARE is demonstrated on simulated data as well as on an experimental yeast dataset.

**Conclusions:**

Using entropy reduction as an objective criterion gives a natural way to tackle both issues of detection and localization simultaneously and to integrate intermediate phenotypic data. We foresee entropy-based strategies as a fruitful research direction for selective phenotyping.

## Background

Quantitative trait mapping aims at finding associations between genetic loci and phenotypes in a population of individuals. As genotyping costs are rapidly decreasing, often phenotyping becomes the limiting factor for mapping genetic traits. Recent techniques of genotyping have enabled access to complete or quasi-complete genotypes, from model organisms like yeast to human individuals [1-3]. Moreover, the number of genotyped individuals has largely increased with genome-wide association studies covering tens of thousands of samples [4]. Yet many phenotypes cannot be measured at this scale, including behavioral traits or susceptibility to drug treatments. The need is therefore increasing for selective phenotyping, *i.e*. the selection of a limited number of samples among a larger library of genotyped samples in order to optimally map a quantitative trait locus (QTL) [5].

Existing methods for selective phenotying methods can be classified into 2 categories (See [6] for a more complete review). The first category aims to maximize the resolution of the mapped loci, typically by maximizing the number of recombination events in the selected set (e.g., [7,8]). The second category has focused on improving the detection power of QTL effects, by selecting a balanced distribution of alleles (e.g., [5], also discussed in [9]). However, no method has been proposed to date that combines both objectives [6].

Here we present SPARE (Selective Phenotyping Approach by Reduction of Entropy), a method that tackles both the issue of QTL effect detection power as well as the issue of locus resolution in a single framework. The problem of selective phenotyping is seen as an experimental design question in which the choice of samples must be made so that the outcome of their phenotyping allows, in expectation, optimal discrimination between the candidate genetic models. Here we follow a Bayesian mapping approach and the objective criterion is the minimization of the entropy of the a posteriori probability of the candidate models. We describe our method for single-causative locus models and fully genotyped populations with only two segregating genotypes, such as backcross, double-haploid, or recombinant inbred lines. An implementation of SPARE in the statistical programming language R [10] is available in Additional File 1 and at http://www.lmb.uni-muenchen.de/tresch/mastermind.html.

## Methods

### Mapping Strategy

In our setting, we have genotyped a set J={1,...,N} of individuals (samples) at a set ℒ={1,...,M} of loci. The genotype of a sample j∈J at a locus λ∈ℒ is denoted by $x_{j,\lambda }\in G$, where G is the set of possible genotypes. The complete genotype information is thus stored in the matrix $x={\left(x_{j,\lambda }\right)}_{j\in J,\lambda ℒ\in ,}$, which is considered as a constant. We assume only two segregating genotypes, such as backcross, double-haploid or recombinant inbred lines and thus G={±1}. Phenotyping a subset of samples S⊂J produces a phenotype vector $y^{S}\in P^{s}$, where P denotes the set of possible phenotypes (typically, phenotypes are considered either binary or continuous). The genotype submatrix of *x *corresponding to the samples *S *is denoted by $x^{S}={\left(x_{s,\lambda }\right)}_{s\in S,\lambda \in L}$. The vector of phenotype measurements *y*^*S *^is the realization of a random variable *Y*^*S*^. If *S *consists of only one individual *s*, we shall write by abuse of notation *y*^*S *^instead of *y*^{*s*} ^and *x*^*s *^instead of *x*^{*s*}^.

The building block of our mapping strategy is the probability of observing a phenotype *y*^*s *^of an individual with genotype *x*^s^, given the QTL locus is λ,

(1) $$\mathrm{Pr}\left(y^{s}|\lambda ,x^{s},\theta \right)$$

Here, *θ *is a suitable set of parameters for the respective model. Assuming observational independence given the genotype, the QTL and the parameters, the likelihood for the locus λ is obtained by summing over the parameters:

(2) $$\mathrm{Pr}\left(y^{S}|\lambda ,x^{S}\right)=\int_{\theta }\mathrm{Pr}\left(y^{S}|\lambda ,x^{s},\theta \right)\mathrm{Pr}\left(\theta |\lambda ,x^{S}\right)d\theta$$

(3) $$=\int_{\theta }\left(\prod_{s\in S}\mathrm{Pr}(y^{s}|\lambda ,x^{s},\theta \right)\mathrm{Pr}(\theta |\lambda ,x^{S})d\theta$$

Specific parameterizations of (1) as well as the choice of appropriate priors Pr(*θ *| λ,*x*^*S*^) in (2) will be discussed later in this section. Note that the individual observations in *y*^*S*^, conditioned only on λ and *x*^*S*^, are not independent in general.

In the Bayesian framework, the mapping process outputs a posterior distribution for the causative locus Λ which is obtained by the Bayesian inversion formula:

(4) $$Pr\left(\lambda |y^{S},x^{S}\right)=\frac{Pr\left(y^{S}|\lambda ,x^{S}\right)\cdot \pi \left(\lambda \right)}{Pr\left(y^{S}|x^{S}\right)}$$

with

(5) $$Pr\left(y^{S}|x^{S}\right)=\underset{\lambda }{\sum }Pr\left(y^{S}|\lambda ,x^{S}\right)\cdot \pi \left(\lambda \right)$$

The locus prior *π*(λ) can for instance be chosen to be uniform.

### Cost function

Similarly to the model discrimination method of Box and Hill [11], we consider the Shannon entropy *H *(Λ) as a measure of the uncertainty about the causative QTL. After having phenotyped a sample subset *S*, the remaining uncertainty about the QTL is:

(6) $$H\left(\Lambda |y^{S},x^{S}\right)=-\underset{\lambda \in L}{\sum }Pr\left(\lambda |y^{S},x^{S}\right)\cdot \log_{2}Pr\left(\lambda |y^{S},x^{S}\right)$$

However, we must make our selection *S *without knowledge of the phenotypic outcome *y*^*S*^, so the best we can do is to minimize the expectation of *H*(Λ|*Y*^*S *^= *y*^*S*^, *x*^*S*^) with respect to *Y*^*S*^|*x*^*S*^. Given a maximum number *n *of individuals to be phenotyped, the task is to select a sample subset S⊂J of size |*S*| = *n *which minimizes

(7) $$cost\left(S\right)=E_{Y^{S}|x^{S}}\left(H\left(\Lambda |Y^{S},x^{S}\right)\right)$$

(8) $$=\int_{y^{S}}H\left(\Lambda |y^{S},x^{S}\right)\cdot Pr\left(y^{S}|x^{S}\right)dy^{S}$$

### Modeling of the genotype-phenotype relation

Three specifications for elementary phenotype distributions (Equation 1) will be discussed here. Generally, let ϵ ∈ {±1} indicate which of the two genotypes is associated with the largest expected phenotypic value. Since this may be unknown, e is a predefined random variable with *P*(ϵ = ±1) = *w*_±1_. The typical choices are either *w*_1 _= 1, *w*_-1 _= 0 for an a priori known genotype-phenotype association, as in typical linkage analysis studies, or $w_{1}=w_{-1}=\frac{1}{2}$ for a completely unknown association. If the phenotype is fully inheritable and perfectly measured, the binary phenotypes *Y*^*s *^deterministically depends upon $x_{\lambda }^{s}$, given the set of parameters *θ *which consists only of the variable ϵ:

(9) $$Y^{s}|\lambda ,x^{s},\theta \sim \delta (Y^{s}=\varepsilon x_{\lambda }^{s})=\{\begin{array}{ll} 1 & \text{if}\;Y^{s}=\varepsilon x_{\lambda }^{s}\\ 0 & \text{otherwise} \end{array}$$

This scenario is called the deterministic case. In the presence of binary measurements with errors, to which we refer as the Bernoulli case, (9) is replaced by a Bernoulli distribution

(10) $$Y^{s}|\lambda ,x^{s},\theta \sim \text{Bernoulli}\left(q_{\varepsilon x_{\lambda }^{s}}\right)$$

with parameters *θ *= (*q*_±1_,ϵ). Equation (2) has to be evaluated for all loci λ. Therefore we make use of conjugate priors for *θ *to obtain a closed form solution of the integral. The conjugate prior is a Beta distribution

$$q_{\varepsilon }\sim Beta\left(\alpha_{\varepsilon },\beta_{\varepsilon }\right)$$

with appropriate hyperparameters *α*_±1_,*β*_±1_. This fully determines the prior if we furthermore assume parameter independence, *i.e*.

(11) $$Pr\left(\theta \right)=Pr\left(\varepsilon \right)Pr\left(q_{1}\right)Pr\left(q_{-1}\right)$$

Finally, we discuss the so-called Gaussian case for quantitative phenotypes, in which the probability (1) is defined as

(12) $$Y^{s}|\lambda ,x^{s},\theta \sim N\left(\mu_{\varepsilon x_{\lambda }^{s}},\sigma^{2}\right)$$

Here, *θ *= (*μ*_±1_, ϵ). We choose conjugate Gaussian priors

(13) $$\mu_{\varepsilon }\sim N\left(\eta_{\varepsilon },\nu^{2}\right)$$

The parameters *σ*^2^, η_±1_, and *ν*^2 ^are considered given and fixed. Again this completely specifies the prior under the assumption of parameter independence, *Pr*(*θ*) = *Pr*(ϵ)*Pr*(*μ*_1_)*Pr*(*μ*_-1_). For all three cases, closed form solutions of equation (2) are derived in the supplements (Additional File 2).

### Incremental selection

We propose a sequential selection strategy that alternates between a selection step and a phenotyping step. This makes use of phenotypic data as they are gathered and is a cost-effective approach for situations where phenotyping is expensive. Let T⊂J be a set of samples disjoint from *S *for which the phenotypes are already known. Equations (4), (5) and (6) hold with *S *∪ *T *in place of *S*. The cost function is now defined given *T *and *y*^*T*^:

(14) $$\begin{array}{ll} cost_{T}\left(S\right) & =E_{Y^{S}|y^{T},x^{T\cup S}}\left(H\left(\Lambda |Y^{S},y^{T},x^{S\cup T}\right)\right)\;\\ & =\int_{y^{S}}H\left(\Lambda |y^{S\cup T},x^{S\cup T}\right)\cdot Pr\left(y^{S}|y^{T},x^{S\cup T}\right)dy^{S}\;\\ & \propto \int_{y^{S}}H\left(\Lambda |y^{S\cup T},x^{S\cup T}\right)\cdot Pr\left(y^{S\cup T}|x^{S\cup T}\right)dy^{S}\;\\ & \; \end{array}$$

By means of cost (14), |*S*| samples are selected and afterwards phenotyped. These selection/phenotyping rounds are iterated until the desired number of individuals is reached. In our applications, we have implemented the case where selection is incremental thus the integration above is done over a single dimension.

#### Remark

It was a discussion about an optimal strategy in the famous recreational Mastermind game that led us to formula (14) and initiated our research. Indeed if we choose loci, genotypes, phenotypes and genotype-phenotype modeling in an abusive way, identifying the hidden target sequence of colors is tantamount to bringing the cost function down to zero. We refer the reader to the supplements for a precise description of the algorithm. There, we implement a SPARE variant that plays the Mastermind game (and does it considerably better than most of us). The Mastermind game has been recently proposed as an educational tool to train biology students to experimental design [12]. Multiple algorithms have been proposed to play Mastermind ([13,14] among many others), including entropy-based approaches [15].

### Simulations

Simulations for two scenarios were done: the *strong linkage *scenario and the *unbalanced allele *scenario. For both scenarios, linkage mapping for a backcross experiment was simulated with three biological replicates of each of the two parental F0 haploid strains and 100 F1 haploid trains. The 100 offspring genotypes were simulated by drawing a number of recombination events given the chromosome length in cM. The positions of the recombination events were drawn uniformly randomly along the chromosome. One unique QTL was drawn from one of the marker positions. The Gaussian model was applied. The fixed parameters were set to *σ*^2 ^= 1,*η*_±1 _= 3, *ν*^2 ^= 4, *w*_±1 _= 0.5. The Gaussian means *μ*_±1 _were drawn from the distributions (13), and the phenotypic values for the parental and the F1 strains were drawn according to (12). In the *strong linkage *scenario, we assumed a single chromosome genotyped at 100 markers equally spaced by 1 cM. No particular filtering is applied on the progeny so that at every marker, the two parental alleles are equally likely to occur. In the *unbalanced allele *scenario, we assumed a single chromosome genotyped at 100 markers equally spaced by 50 cM. Two alleles have low frequencies (20% in expectation) in the population: the causative allele and another randomly drawn one. This is obtained by first generating a much larger F1 population and then taking a random subset of 100 individuals weighting the probability to choose each individual according to its genotype for the two low frequency alleles. This procedure generates a population in which linkage is respected and the two low frequency alleles have in expectation a 20% frequency.

On these datasets, SPARE was initialized given the observation of the parental phenotypes. This setting matches the typical situation in which a cross is used to map a phenotypic difference observed between parental strains. SPARE, however, does not require initialization with parental phenotypes. We iterated the selection step until 30 out of the 100 F1 strains were included. For each selection size, we selected the samples by optimizing the MMA criterion using a two-step implementation [5] (a greedy search followed by greedy swaps). Optimization of the SBBL criterion was done in the same fashion. For each of the three methods, one obtains a series of 30 selection sets of size 1 to 30. The selection of SPARE consists of an increasing chain of sets, whereas the result of the other methods does not necessarily do so. We let MMA and SSBL run in a non-incremental fashion as these methods were not designed for this approach and, in contrast to SPARE, do not take the phenotypic information into account.

## Results

### Performance for detection and for resolution of QTLs

We compared our method to two methods representative of the two current selective phenotyping strategy families, *i.e*. one optimizing the resolution power and one the detection power. On the one side, the SSBL method [8] minimizes the Sum of the Squared Bin Lengths, where a bin is defined as a genomic interval within which there are no crossovers in any individual and that is bounded on either side by a crossover in at least one individual [16]. Minimizing the SSBL favors selections with evenly distributed crossovers along the genome. It therefore facilitates discriminating between markers in linkage and thus narrowing down QTL intervals. Methods focusing on resolution power are particularly advantageous when linkage is strong between alleles. On the other side, MMA, for Minimum Moment of Aberrations [5], maximizes the genotype dissimilarities at every marker in the selection thereby increasing the detection power. For two segregating genotypes, this favors balanced representation of the two alleles at each marker. This can be particularly advantageous for example in situation where the causative allele has a low frequency in the population, because it will enrich for carriers of the allele in the selection and facilitate the detection of its effect.

Simulations for two scenarios were done. In the *strong linkage *scenario, alleles at each marker are equally distributed but linkage is strong; therefore, resolution power is the main issue. In the *unbalanced allele *scenario, linkage is weak but the causative allele has a low frequency; thus, detection power is the main challenge (see Methods for details). Unbalanced allele frequencies are common in outbred populations. As in our simulation, unbalanced allele frequency can also occur in recombinant inbred line panels if combinations of alleles are lethal or lead to phenotypes incompatible with the assay of interest. The whole procedure was repeated 100 times for each scenario. In our formulation, performing QTL mapping amounts computing the posterior distribution over the candidate loci. We used the Kullback-Leibler distance to estimate how close the posterior distribution for a particular selection is to the posterior obtained when phenotypes of the whole population are taken into account. This measure allows us to compare the performance of different methods based on the selection they propose.

**Figure 1:**
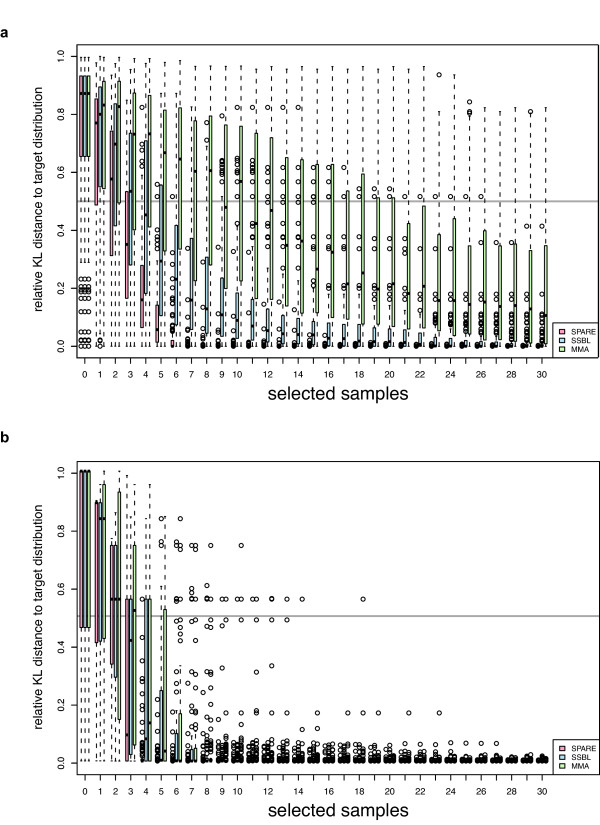
**Benchmarking of SPARE, SSBL and MMA on the strong linkage (a) and on the unbalanced allele scenario (b)**. The posterior distribution obtained when phenotyping all available individuals is considered as the target distribution. The relative Kullback-Leibler distance (KL distance, Equation 6) to target distribution (y-axis) is the KL distance of a given posterior distribution to the target distribution divided by the KL distance of the initial posterior distribution (parental strains only) to the target distribution. Each boxplot summarizes the performance of 100 simulation runs for SPARE (pink), SSBL (blue) and MMA (green). Boxplots are grouped by selection sample size and selection method.

**Figure 2:**
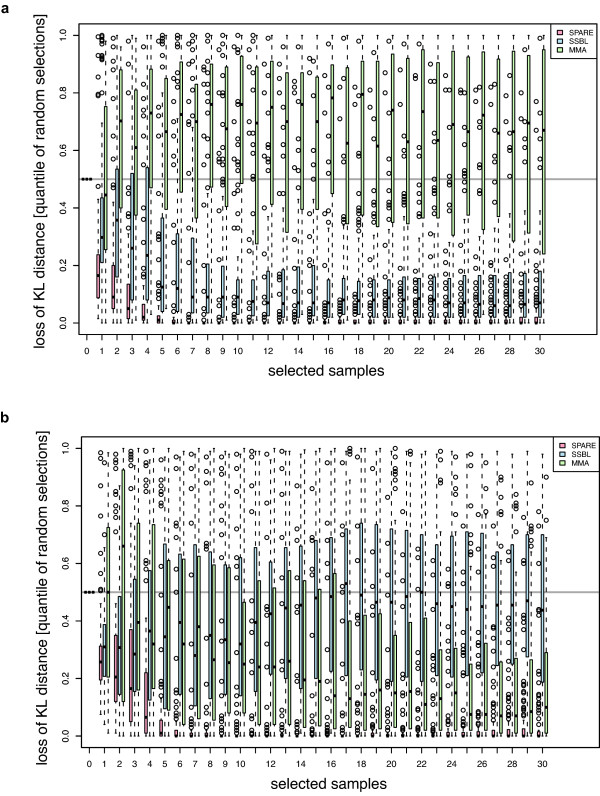
**Benchmarking of SPARE, SSBL and MMA on the strong linkage (a) and on the unbalanced allele scenario (b) against random selections**. As for Figure 1, the posterior distribution obtained when phenotyping all available individuals is considered as the target distribution. Each boxplot shows the Kullback-Leibler distance (KL distance, Equation 6) from the posterior of each selection to the target distribution for 100 simulation runs. Boxplots are grouped by selection sample size and selection method. For each selection size, the KL distance is expressed in terms of quantile within 100 random selections for SPARE (pink), SSBL (blue) and MMA (green). Horizontal grey line marks the median KL within the random selections.

As expected SSBL performs better than MMA in the strong linkage scenario (Figure 1a and Figure 2a), whereas MMA performs better in the unbalanced allele scenario (Figure 1b and Figure 2b). In both scenarios, however, SPARE is the most effective method. Indeed, SPARE outperforms both SSBL and MMA, reaching a posterior close to the optimal one much earlier than the other methods (on average after 6 further F1 strains for the strong linkage scenario, while SSBL requires 20 F1 strains on average for similar QTL mapping accuracy, Figure 1a). We further compared all three methods to random selections. For every selection size, we randomly picked a set of the given size 100 times and expressed the performance of each method as its rank within these 100 random draws (Figure 2). MMA does not improve over random selections when applied to the strong linkage scenario, with a median ranking among random selections greater than 50% (Figure 2a). SSBL performed slightly better than random in the unbalanced allele scenario (with a median ranking at typically 40%, first quartile rank at 20%, and third quartile rank at about 70% across all selection sizes). SPARE, in contrast, shows consistent improvements over random selections in both settings. These simulations show that SPARE performs well, both in situations where detection is the main challenge as well as in situations where resolution is the issue.

### Advantage of incremental phenotyping

**Figure 3:**
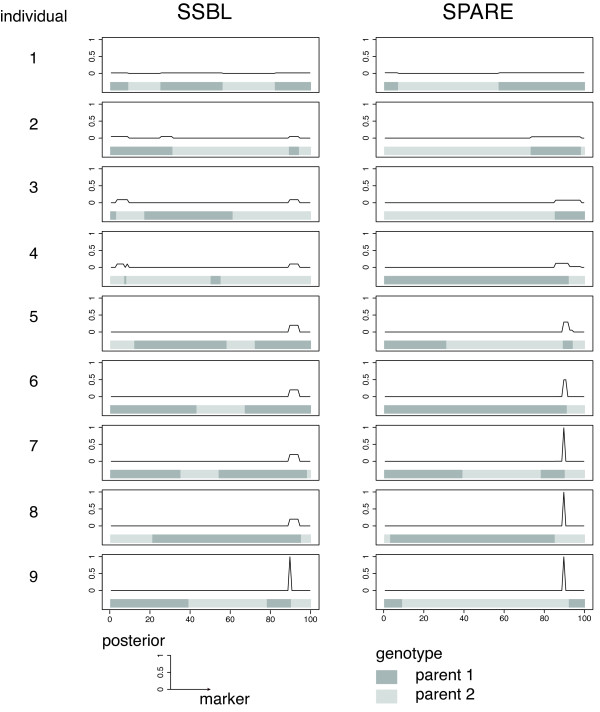
**Comparison of SSBL and SPARE for incremental phenotyping**. The first 9 selected individuals of SBBL (left) and SPARE (right) for one run of the strong linkage simulation scenario. Selected individuals are phenotyped one after the other sequentially (from top to bottom). The horizontal bars show the genotype of each individual along the 100 markers (dark grey genotype of parent 1, light grey genotype of parent 2). The updated posterior probability after phenotyping the selected individual is displayed above. SPARE, which can make use of previous phenotypes, selects individuals with crossovers lying in the current QTL interval, thereby narrowing it down faster to a single marker.

SPARE can integrate the information of phenotyped individuals, giving SPARE an advantage over previous methods. This can be well illustrated in the case of the strong linkage scenario, and holds true for the unbalanced allele scenario. Figure 3 shows a representative selection series of SPARE versus SSBL on the same dataset. The true causative locus was at position 90. The two methods are compared for a selection of 9 individuals. The set of SSBL as a whole provides approximately evenly distributed crossovers along the chromosome. However, because SSBL does not make use of phenotypic information, the selection of individual at each step is disconnected from the actual most likely candidate loci. In contrast, SPARE selects individual with crossovers relevant to the current posterior, enabling us to pinpoint the causative locus in only 7 steps (vs. 9 for SSBL).

### Application to a yeast dataset

We then applied SPARE to a linkage analysis dataset in yeast [17]. This dataset contains 184 meiotic recombinants genotyped at high resolution [1] and were phenotyped for growth in presence of arsenate (a binary trait). Using the phenotypic information of all 184 segregants, the original study identified and validated a single locus as determinant, the gene *PHO84*. SPARE, SSBL and MMA were run on this dataset, restricting for computational efficiency reasons to 314 markers regularly spaced at 100 cM distance. Different prior parameters were tried. The optimal prior, which takes as pseudo-counts of the Beta distributions the counts observed in the total population of 184 segregants: *q*_1 _~ *Beta*(76, 17) and *q*_-1 _~ *Beta*(12, 79), the uniform prior, and a strong prior but with an association to the locus closer to random (60%): *q*_1 _~ *Beta*(60, 40) and *q*_-1 _~ *Beta*(40, 60). The genotype-phenotype association is known here from the parental phenotypes and we therefore set *w*_1 _= 1 and *w*_-1 _= 0. As for the simulations, the Kullback-Leibler distance to the posterior estimated on the whole population was taken as a measure of the accuracy of the mapping for any selection.

**Figure 4:**
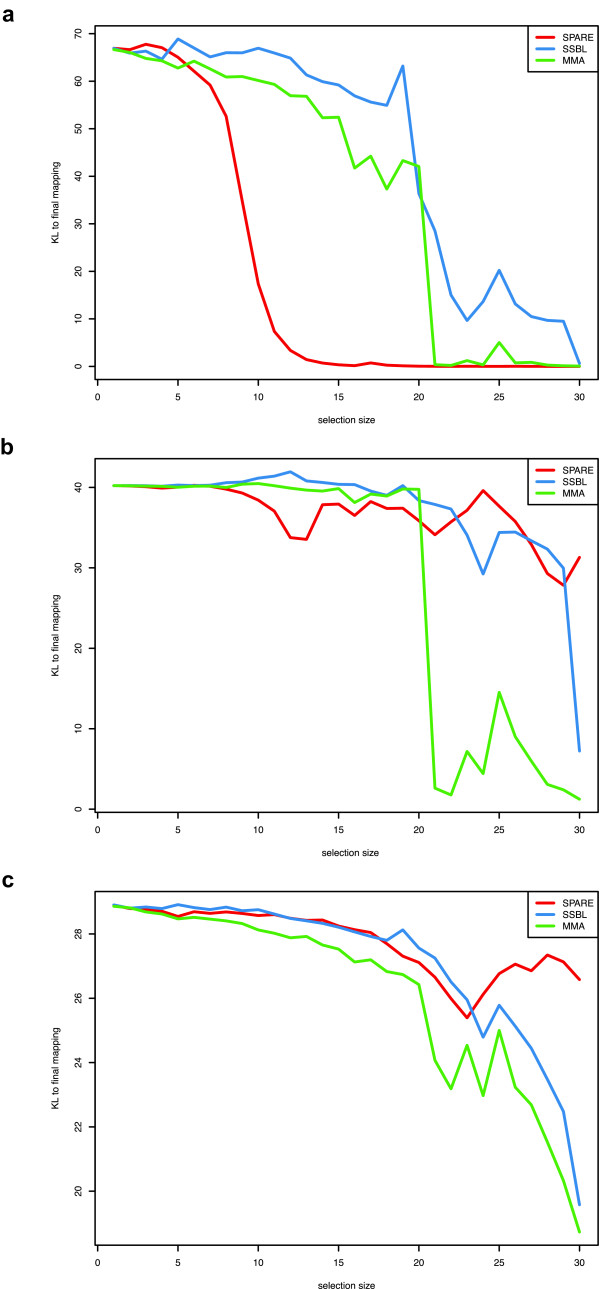
**Benchmark on the yeast dataset**. Kullback-Leibler distance to final mapping (y-axis) versus selection size (x-axis) for three selection methods, SPARE (red), SSBL (blue) and MMA (green) with different prior distributions: optimal prior corresponding to the counts observed on the whole population (a), uniform prior (b), strong prior with an association closer to random (c).

SPARE outperformed SSBL and MMA when run with the optimal prior, identifying the causative locus after phenotyping 12 to 15 segregants (Figure 4). However, it performed less accurately with the strong prior close to random associations as well as with the uniform prior. Hence, at least on this particular dataset, the prior seems to play a role in the performance of SPARE.

## Discussion

We introduced SPARE, a selective phenotyping method based on Bayesian model discrimination that tackles both resolution and detection issues simultaneously. The objective function we minimize is an expected entropy which favors both a balanced selection of genotypes at all loci as well as evenly distributed crossovers across the genome. This formulation provides a natural way to deal with the issue of resolution: loci in linkage are seen as alternative competing models. Interestingly, linkage between neighboring loci does not have to be explicitly modeled. Indeed, two loci in linkage lead to similar likelihood. Moreover, our approach is able to make use of prior phenotype information and thereby proposes selections that enable the rapid reduction of QTL intervals. Simulations showed that SPARE can provide, on average, a more efficient selection strategy than previous methods. Application to a linkage analysis dataset in yeast showed that SPARE can propose, with appropriate priors, selections that enable a faster mapping of the QTL.

Even though SPARE is a Bayesian method, one should note that just like other selective phenotyping procedure, its application is independent of the method employed to perform QTL mapping, which can be frequentist. It is worth noting however that comparing QTL-mapping results such as LOD score curves for different selections is a not a well-defined task. Indeed, the objective of QTL mapping is not only to detect the causative locus, for example with a significant LOD score, but also to have a narrow confidence interval on its location. One contribution of the present study is to propose the entropy of the posterior to quantify how certain, *i.e*. how 'high and well-peaked', the mapping is. It relies on a Bayesian framework.

SPARE requires a prior distribution of the trait given the causative locus. The application to the yeast dataset showed that sensitivity to this prior distribution mattered. SPARE could only give the best results when applied with the prior distribution close to the optimal one. This is currently a weak point of the method.

Two extensions could broaden the applicability of the current framework: First, the restriction to the case of simple traits with a single causative locus may be dropped. One would like to detect complex traits, *i.e*. those involving multiple causative loci. Second, a selection step that chooses more than one sample at a time is desirable as phenotyping individuals in small batches might be more cost-effective than for one individual at a time. However, both improvements are computationally prohibitive if our cost function is used without modification, because then the task turns into a combinatorial optimization problem. Instead, efficient approximations must be developed to cope with the explosion in the number of hypotheses (Equation 5), and to solve the integration step in high dimensional space (Equation 8 and 14). The analogy to the Mastermind game suggests directions to a possible solution. For instance Cotta and colleagues [18] do not consider all possible hypotheses in the Mastermind game; they evaluate a representative subset of it, and this subset is evolved during the course of the game. Alternatively, the integration required in Equation 8 might be replaced by a sampling method. Another alternative is to change the cost function. It has been shown that Mastermind strategies that simply maximize the number of possible answers do surprisingly well [19]. An analogous phenotyping strategy would be promising even if more than one locus was associated with the phenotype. However, it was unclear to us how to transfer that approach to continuous phenotypes.

Our simulations also showed that random selections are actually performing reasonably well compared to standard methods in a linkage analysis approach, with balanced allele frequencies. While unexpected, this observation can be explained. Indeed, optimal selections are selections that are simultaneously balanced at each polymorphism and with evenly distributed break points along the genome. The natural process of recombination actually promotes this situation. Random draws thus typically show these desired features.

Recently, the use of Fisher's information to evaluate experimental designs in the context of QTL mapping, including selective phenotyping has been discussed [9]. In contrast to our method, Sen and colleagues assume the QTL locations given and use criteria based on Fisher's information to compare experimental designs. Hence, while the method of Sen and collaborators allows dealing with multiple-loci models, it does not address the question of resolution. The relationships between Fisher's information and Shannon entropy criteria in this context constitute an interesting direction of research.

## Conclusion

SPARE is a Bayesian model discrimination approach to perform selective phenotyping. Using entropy reduction as an objective criterion enabled tackling both issues of detection and localization jointly for the first time and integrating intermediate phenotypic data. Altogether, entropy-based strategies appear as a promising family of approaches for the question of selective phenotyping.

## Authors' contributions

JG and AT conceived the research. JG, ME and AT devised and implemented the algorithm. JG and AT wrote the manuscript. All authors read and approved the final manuscript.

## Supplementary Material

Additional file 1 **SPARE source code**. A zip-compressed directory containing R source code and example data. See file README.txt in there for more details. Click here for file

Additional file 2 **Supplementary Information**. Click here for file
