# Supplementary material for: Selective Phenotyping, Entropy Reduction, and the Mastermind game
Source: BMC Bioinformatics. 2011 Oct 20;12:406. doi: 10.1186/1471-2105-12-406 (PMC3258278; doi:10.1186/1471-2105-12-406)
Supplement: Additional file 2 — Supplementary Information. [file 1471-2105-12-406-S2.PDF]

Supplementary Information to the article 'Selective Phenotyping, Entropy Reduction and the Mastermind Game'

Julien Gagneur*[1], Markus Elze[2], Achim Tresch[3]

[1]European Molecular Biology Lab, 69122 Heidelberg, Germany
[2]Johannes Gutenberg-University, 55099 Mainz, Germany
[3]Gene Center, Ludwig-Maximilians-University, 81377 Munich, Germany

Email: Julien Gagneur*- julien.gagneur@embl.de; Markus Elze - m.elze@warwick.ac.uk; Achim Tresch - tresch@lmb.uni-muenchen.de;

*Corresponding author

1 Likelihood functions

In this section, we derive closed form solutions for the likelihood function (Equation 2 in the article). Marginalizing over θ, we obtain

$$\Pr(y^S \mid \lambda, x^S) = \int_\theta \left(\Pr(y^s \mid \lambda, x^s, \theta) \Pr(\theta \mid \lambda, x^s)\right) \tag{1}$$

We derive closed-form solutions in all three (deterministic, Bernoulli, Gaussian) cases.

1.1 The deterministic case

The parameters are $\theta = (\epsilon)$.

$$\begin{aligned}
\Pr(y^S \mid \lambda, x^S) &= \int_\theta \left(\prod_{s\in S} \Pr(y^s \mid \lambda, \theta, x^s)\right) \Pr(\theta \mid \lambda, x^S) d\theta \\
&= \sum_{\epsilon=\pm1} \left(\prod_{s\in S} \delta(y^s = \epsilon x^s_\lambda)\right) Pr(\epsilon) \\
&= \begin{cases} w_1 & \text{if } y^S = x^S_\lambda \\ w_{-1} & \text{if } y^S = -x^S_\lambda \\ 0 & \text{otherwise} \end{cases}
\end{aligned} \tag{2}$$

If we furthermore assume a uniform prior $\pi(\lambda) = \frac{1}{M}$, we can even derive a formula for the entropy and the cost function: Let $n(y^S) = \#\left\{\lambda \mid y^S = x^S_\lambda\right\}$ and $D(y^S) = w_1 n(y^S) + w_{-1} n(-y^S)$. Then using Equations 4 and 5 of the article, we obtain

$$\begin{aligned} Pr(y^S \mid x^S) &= \frac{1}{M}\sum_{\lambda} Pr(y^S \mid \lambda, x^S) \\ &= \frac{1}{M}\left(w_1 n(y^S) + w_{-1} n(-y^S)\right) \\ &= \frac{D(y^S)}{M} \end{aligned} \tag{3}$$

$$\begin{aligned} Pr(\lambda \mid y^S, x^S) &= \frac{M}{D(y^S)} \cdot \begin{cases} w_1/M & \text{if } y^S = x_\lambda^S \\ w_{-1}/M & \text{if } y^S = -x_\lambda^S \\ 0 & \text{otherwise} \end{cases} \\ &= \begin{cases} w_1/D(y^S) & \text{if } y^S = x_\lambda^S \\ w_{-1}/D(y^S) & \text{if } y^S = -x_\lambda^S \\ 0 & \text{otherwise} \end{cases} \end{aligned} \tag{4}$$

this leads to

$$H(\Lambda \mid y^S, x^S) = -\sum_{\lambda} Pr(\lambda \mid y^S, x^S) \cdot \log_2 Pr(\lambda \mid y^S, x^S) \tag{5}$$

$$= \begin{cases} 0 \quad \text{if } \; D(y^S) = 0 \\ -\sum_{j\in\{\pm 1\}} n(jy^S) \cdot \frac{w_j}{D(y^S)} \log_2 \frac{w_j}{D(y^S)} \quad \text{else} \end{cases}$$

The cost function now becomes

$$\begin{aligned} cost(S) &= \sum_{y^S} H(\Lambda \mid y^S, x^S) \cdot Pr(y^S \mid x^S) \\ &= -\frac{1}{M} \sum_{\substack{y^S, \\ D(y^S)>0}} \left(w_1 n(y^S) \log_2 \frac{w_1}{D(y^S)} + w_{-1} n(-y^S) \log_2 \frac{w_{-1}}{D(y^S)} \right) \end{aligned} \tag{6}$$

The summation is not over all the $2^{\#S}$ possible values for y^S, but only over those vectors y^S for which $D(y^S) > 0$, which can only happen if y^S equals one of the genotype vectors x_λ^S or $-x_\lambda^S$, $\lambda = 1, ..., M$. Thus the sum (6) includes at most $2M$ terms, which for larger S is a substantially smaller number than $2^{\#S}$. In practice, the unique genotype vectors x_λ^S are first computed as unique column-wise entries of the genotype matrix x^S.

Now suppose we have already phenotyped a nonempty subset T and we would like to select the next set S

of individuals. Then we need to evaluate

$$
\begin{aligned}
& p(y^S \mid y^T, x^{S\cup T}) = \\
&= \sum_{\lambda} p(y^S \mid \lambda, y^T, x^{S\cup T}) p(\lambda \mid y^T, x^{S\cup T}) \\
&= \sum_{\lambda,\epsilon} p(y^S \mid \epsilon, \lambda, y^T, x^{S\cup T}) p(\epsilon \mid \lambda, y^T, x^S, x^T) p(\lambda \mid y^T, x^{S\cup T}) \\
&= \sum_{\lambda} p(\lambda \mid y^T, x^T) \begin{cases} p(y^S \mid \epsilon = 1, \lambda, y^T, x^{S\cup T}) & \text{if } y^T = x^T_\lambda \\ p(y^S \mid \epsilon = -1, \lambda, y^T, x^{S\cup T}) & \text{if } y^T = -x^T_\lambda \\ 0 & \text{otherwise} \end{cases} \\
&= \sum_{\lambda, y^T = x^T_\lambda} p(\lambda \mid y^T, x^T) \cdot \delta(y^S = x^S) + \sum_{\lambda, y^T = -x^T_\lambda} p(\lambda \mid y^T, x^T) \cdot \delta(y^S = -x^S) \\
&= \sum_{\lambda, y^{T\cup S} = x^{T\cup S}_\lambda} p(\lambda \mid y^T, x^T) + \sum_{\lambda, y^{T\cup S} = -x^{T\cup S}_\lambda} p(\lambda \mid y^T, x^T) \\
&\overset{(4)}{=} \begin{cases} \frac{w_1 n(y^{T\cup S}) + w_{-1} n(-y^{T\cup S})}{D(y^T)} & \text{if } D(y^T) > 0 \\ 0 & \text{if } D(y^T) = 0 \end{cases}
\end{aligned}
\tag{7}
$$

Equations 5 and 7 allow the fast calculation of the cost as

$$
\begin{aligned}
cost_T(S) &= E_{Y^S|x^S}(\, H(\Lambda \mid Y^S, x^S)\,) \\
&= \sum_{y^S} Pr(y^S \mid y^T, x^{S\cup T}) \cdot H(\Lambda \mid y^{S\cup T}, x^{S\cup T}) \\
&= \sum_{y^S, D(y^{S\cup T})>0} Pr(y^S \mid y^T, x^{S\cup T}) \cdot H(\Lambda \mid y^{S\cup T}, x^{S\cup T})
\end{aligned}
\tag{8}
$$

1.2 The Bernoulli case

The parameters are $\theta = (q_1, q_{-1}, \epsilon)$. For given y^S, v, let $n_{\epsilon_1,\epsilon_2} = \#\{s \in S \mid y^s = \epsilon_1,\ v = \epsilon_2\}$ be the crosstable of the phenotype vector y^S and the genotype vector v, and let $B(\alpha, \beta) = \frac{\Gamma(\alpha)\Gamma(\beta)}{\Gamma(\alpha+\beta)}$ the Beta function. Define

$Q(y^s; v)$ as the probability of observing y^s given that the genotype for the causative locus is v:

$$
\begin{aligned}
Q(y^S; v) &= \int_\theta \left(\prod_{s\in S} \Pr(y^s \mid x^s_{\lambda_{\text{true}}} = v, \theta, x^s) \right) \Pr(\theta \mid \lambda, x^S) \qquad (9)\\
&= \sum_\epsilon \int_{q_1} \int_{q_{-1}} \left(\prod_{s\in S} \begin{cases} q_{\epsilon v} & \text{if } y^s = 1 \\ 1 - q_{\epsilon v} & \text{if } y^s = -1 \end{cases} \right) \cdot B(\alpha_1, \beta_1)^{-1} q_1^{\alpha_1 - 1} (1 - q_1)^{\beta_1 - 1} \\
&\quad \cdot B(\alpha_{-1}, \beta_{-1})^{-1} q_{-1}^{\alpha_{-1}-1} (1 - q_{-1})^{\beta_{-1}-1} w_\epsilon \\
&= B(\alpha_1, \beta_1)^{-1} B(\alpha_{-1}, \beta_{-1})^{-1} \sum_\epsilon \int_{q_1} q_1^{n_{1,\epsilon}+\alpha_1-1} (1 - q_1)^{n_{-1,\epsilon}+\beta_1-1} \\
&\quad \cdot \int_{q_{-1}} q_{-1}^{n_{1,-\epsilon}+\alpha_{-1}-1} (1 - q_{-1})^{n_{-1,\epsilon}+\beta_{-1}-1} w_\epsilon \\
&= \sum_{\epsilon=\pm 1} \frac{B(\alpha_1 + n_{1,\epsilon}, \beta_1 + n_{-1,\epsilon})}{B(\alpha_1, \beta_1)} \frac{B(\alpha_{-1} + n_{1,-\epsilon}, \beta_{-1} + n_{-1,-\epsilon})}{B(\alpha_{-1}, \beta_{-1})} w_\epsilon
\end{aligned}
$$

In other words, if we phenotype the sample set S , given the QTL is λ, we obtain

$$Pr(y^S \mid \lambda) = Q(y^S; x^S_\lambda)$$

1.3 The Gaussian case

The parameters are $\theta = (\mu_1, \mu_{-1}, \epsilon)$. Again, fix y^S, λ for the moment. Let $\tau = \sigma^{-2}$, $\tau' = \nu^{-2}$. Let $S_\epsilon = \{s \mid x^s_\lambda = \epsilon\}$, $\epsilon = \pm 1$. For the case of unknown association, the likelihood function is

$$
\begin{aligned}
\Pr(y^S \mid \lambda, x^S) &= \int_\theta \left(\prod_{s\in S} \Pr(y^s \mid \lambda, \theta, x^s) \right) \Pr(\theta \mid \lambda, x^S) \qquad (10)\\
&= \sum_\epsilon \int_{\mu_1} \int_{\mu_{-1}} \left(\prod_{s\in S} \Pr(y^s \mid \lambda, \theta, x^s) \right) Pr(\mu_1) Pr(\mu_{-1}) Pr(\epsilon) \\
&= \sum_\epsilon \int_{\mu_1} \int_{\mu_{-1}} \left(\prod_{s\in S} \frac{1}{\sigma\sqrt{2\pi}} \exp\left[-\frac{\tau}{2} (y^s - \mu_{\epsilon x^s_\lambda})^2 \right] \right) \\
&\quad \cdot \left(\frac{1}{\nu\sqrt{2\pi}} \exp\left[-\frac{\tau'}{2} (\mu_1 - \eta_1)^2 \right] \right) \\
&\quad \cdot \left(\frac{1}{\nu\sqrt{2\pi}} \exp\left[-\frac{\tau'}{2} (\mu_{-1} - \eta_{-1})^2 \right] \right) w_\epsilon \\
&= \frac{1}{\nu^2 \sigma^n \sqrt{2\pi}^{n+2}} \sum_\epsilon \int_{\mu_1} \exp\left[-\frac{\tau}{2} \sum_{s\in S_\epsilon} (y^s - \mu_1)^2 - \frac{\tau'}{2} (\mu_1 - \eta_1)^2 \right] \\
&\quad \cdot \int_{\mu_{-1}} \exp\left[-\frac{\tau}{2} \sum_{s\in S_{-\epsilon}} (y^s - \mu_{-1})^2 - \frac{\tau'}{2} (\mu_{-1} - \eta_{-1})^2 \right] w_\epsilon
\end{aligned}
$$

Sorting terms in the exponential by powers of μ_1 resp. μ_{-1}, we make use of the textbook formula

$$\int_x \exp(-\frac{1}{2} \left(ax^2 + bx + c\right)) = \frac{\sqrt{2\pi}}{\sqrt{a}} \exp(-\frac{1}{2} \left(c - \frac{b^2}{4a} \right)) \qquad (11)$$

to obtain a closed form of the integrals

$$\int_{\mu_d} \exp\left[-\frac{\tau}{2}\sum_{s\in S_{(d\epsilon)}}(y^s-\mu_d)^2-\frac{\tau'}{2}(\mu_d-\eta_d)^2\right]$$

$$=\frac{\sqrt{2\pi}}{\sqrt{a_{(d\epsilon)}}}\exp\left[-\frac{1}{2}(c_{d,\epsilon}-\frac{b_{d,\epsilon}^2}{4a_{(d\epsilon)}})\right] \tag{12}$$

for $d,\epsilon=\pm1$. Here, $a_{(d\epsilon)}=\tau|S_{(d\epsilon)}|+\tau'$, $b_{d,\epsilon}=-2(\tau\sum_{s\in S_{(d\epsilon)}}y^s+\tau'\eta_d)$ and $c_{d,\epsilon}=\tau\sum_{s\in S_{(d\epsilon)}}(y^s)^2+\tau'\eta_d^2$. Letting $a=\sqrt{a_{1,\epsilon}a_{-1,\epsilon}}=\sqrt{(\tau|S_1|+\tau')(\tau|S_{-1}|+\tau')}$ and $c=c_{1,\epsilon}+c_{-1,\epsilon}$, equation (10) can be written as

$$\Pr(y^S\mid\lambda,x^S)=\frac{1}{a\nu^2\sigma^n\sqrt{2\pi}^n}\sum_{\epsilon=\pm1}\exp\left[-\frac{1}{2}(c-\frac{b_{1,\epsilon}^2}{4a_\epsilon}-\frac{b_{-1,\epsilon}^2}{4a_{-\epsilon}})\right]\cdot w_\epsilon \tag{13}$$

2 How to play Mastermind

First, let us shortly recall the rules of the Mastermind^{TM} game. It is essentially a one-player game in which the player tries to guess a hidden (target) sequence of coloured pins in a minimum number of attempts. A target sequence consists of N coloured pins (usually $N=4$ or 5) which are drawn from a set of C colours (ususally $C=6$, 7 or 8). Drawing can be done with or without replacement, the subsequent considerations apply to both variants of the game. The player tries to guess the hidden sequence in a minimum number of attempts. One attempt consists of writing down a candidate (query) sequence, and receiving a pair of numbers as an answer. The first of these numbers tells how many pins in the query sequence exactly match the corresponding pins in the target sequence, and the second number indicates how many of the non-matching pins in the query sequence have a colour that occurs in the target sequence. Again, the algorithm proposed here is works correctly, no matter whether a query sequence is required to contain pairwise different colours, or if it may consist of an arbitrary sequence of pins.

The target sequence identification task can be cast as a mapping problem. Let the locus set $\mathcal{L}$ as well as the sample set $\mathcal{S}$ equal the set of all possible query sequences. The genotype $x_{s,\lambda}$ of sample $s\in\mathcal{S}$ at locus $\lambda\in\mathcal{L}$ is the answer the player would get in response to the query s if λ were the true target sequence. Hence the genotype set $\mathcal{G}$ consists of all pairs of non-negative integers that might be obtained as an answer. Moreover, let the phenotype set $\mathcal{P}$ equal the genotype set $\mathcal{G}$. The "phenotyping process" of sample s returns an answer $y^s\in\mathcal{P}$. The elementary likelihood function then corresponds to that of the deterministic case for $\epsilon_1=1$):

$$Y^s\mid\lambda,x^s\sim\delta(Y^s=x^s_\lambda) \tag{14}$$

We implemented the incremental learning strategy for the Mastermind game as an R program [1]. The computer tries to guess the hidden sequence which the user has figured out. The software is available at

http://www.lmb.uni-muenchen.de/tresch/mastermind.html. Supplementary Figures 1 and 2 show a sample run of our Mastermind algorithm.

```
*** The Mastermind Game ***
The present version does not allow repeated colors in the target- or
query sequence

Please enter the number of colors used (followed by <enter>): 8
Please enter the number of pins in the target sequence (followed by
<enter>): 4

Please choose a hidden target sequence ofl ength 4 from the alphabet
{ A,B,C,D,E,F,G,H      } , without repetitions
Then type <enter> to start the game

Round #1
My query sequence is (A C H B) How many exact matches does it
contain? 1
How many additional inexact matches does it contain? 1
There are 288 possible combinations left

Round #2
My query sequence is (A C D E)
How many exact matches does it contain? 0
How many additional inexact matches does it contain? 2
There are 60 possible combinations left

Round #3
My query sequence is (D F H C)
How many exact matches does it contain? 1
How many additional inexact matches does it contain? 1
There are 12 possible combinations left

Round #4
My query sequence is (B A H D)
How many exact matches does it contain? 2
How many additional inexact matches does it contain? 0
There are 1 possible combinations left

Round #5
My query sequence is (E A H F)
How many exact matches does it contain? 4

I needed 5 attempts to find your hidden sequence.
The history of the game is shown nearby in the graphics window.
```

Figure 1: A sample run of the Mastermind algorithm. Here, 8 colours and 4 pins were used, and target- and query sequences are without repetitions. Our hidden sequence was (E,A,H,F).

References

1. R Development Core Team: *R: A Language and Environment for Statistical Computing*. R Foundation for Statistical Computing, Vienna, Austria 2011, [http://www.R-project.org]. [ISBN 3-900051-07-0].

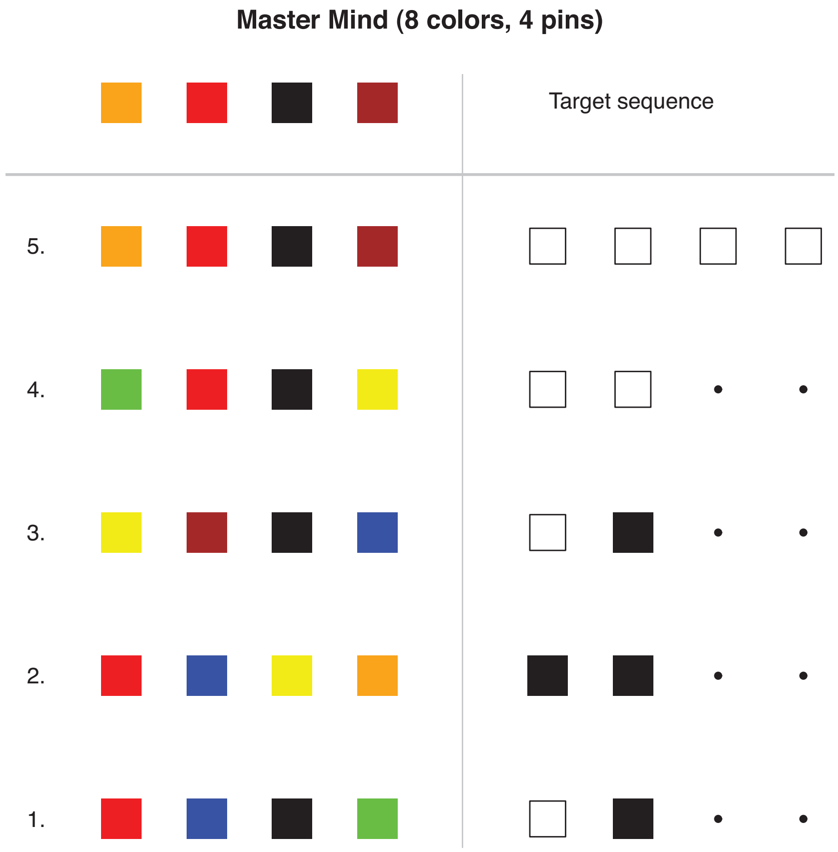

Figure 2: Graphical visualization of the history of the game played in Figure 1.
